# Assessment of 2022 European LeukemiaNet risk classification system in real‐world cohort from China

**DOI:** 10.1002/cam4.6696

**Published:** 2023-12-14

**Authors:** Enbo Chen, Changqing Jiao, Jian Yu, Yu Gong, Duo Jin, Xiaoyu Ma, Jianling Cui, Zhonghui Wu, Junjie Zhou, Haixia Wang, Bobing Su, Jian Ge

**Affiliations:** ^1^ Department of Hematology The First Affiliated Hospital of Anhui Medical University Hefei Anhui China; ^2^ Department of Hematology Chaoyang Hospital Huainan Anhui China; ^3^ Department of Hematology Taihe County People's Hospital Fuyang Anhui China

**Keywords:** acute myeloid leukemia, European LeukemiaNet, FLT3‐ITD mutations, real‐world, risk classifications

## Abstract

**Background:**

The European LeukemiaNet (ELN) risk classification system for acute myeloid leukemia (AML) patients has been used worldwide. In 2022, the ELN risk classification system modified risk genes including CEBPA mutation status, myelodysplasia‐related (MR) gene mutations and internal tandem duplications of FLT3 (FLT3‐ITD).

**Methods:**

We include newly diagnosed de novo AML patients at our center from January 2017 to December 2021, regardless of the further treatment received. Clinical data and date of survival were included. Survival analysis were performed using the Kaplan–Meier method, and the log‐rank test was used to compare survival between different risk groups.

**Results:**

We include 363 newly diagnosed de novo AML patients from 2017 to 2021 to assess the accuracy of the ELN risk classification system. Their survival results show that the ELN‐2022 risk classification system is not superior to the ELN‐2017 version; for patients with FLT3‐ITD mutations but without FLT3 inhibitor treatment, their survival is similar to the ELN‐2022 adverse risk group. The ELN‐2022 risk classification system cannot accurately clarify ECOG performance status (PS) 2–4 patients, especially in the ELN‐2022 favorable risk group.

**Conclusion:**

The ELN‐2022 risk stratification system may not be appropriate for patients unable to receive intensive therapy or FLT3 inhibitor; more real‐world data is needed to straify patients with worse ECOG PS and inferior intensive therapy.

## Graphical abstract

In 2022, the European LeukemiaNet (ELN) risk classification system for acute myeloid leukemia patients modified risk genes including CEBPA mutation status, myelodysplasia‐related gene mutations and internal tandem duplications of FLT3 (FLT3‐ITD). We include 363 newly diagnosed de novo AML patients at our center from 2017 to 2021 to assess the accuracy of the ELN risk classification system. Their overall survival results show that the ELN‐2022 risk classification system may not be superior to the ELN‐2017 version in our real‐world cohort.

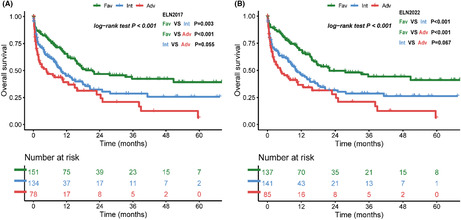

## 1 INTRODUCTION

Acute myeloid leukemia (AML) is a clonal malignancy characterized by proliferation of myeloid blast with expansion and block in differentiation, resulting in inefficient normal hematopoiesis, life‐threatening cytopenia and transfusion dependency.^1^ In 2010, an international expert panel in the name of the European LeukemiaNet (ELN) created a new standardized risk classification system based on patients' cytogenetic and molecular genetic data, which was simplified and adapted in 2017.^2^, ^3^, ^4^ The ELN‐2017 risk classification system, which classifies AML patients as favorable, intermediate and adverse groups, has been validated effective in intensively treated AML patient cohorts and is widely used in clinical practice worldwide.^5^, ^6^, ^7^, ^8^ In consideration of new clinical trial reports and widely‐used measurable residual disease (MRD), the ELN updated AML guidelines in 2022 and adjusted the risk classification. First, based on clinical trials showing similar outcomes between patients with low and high internal tandem duplications of FLT3 (FLT3‐ITD) allelic ratios,^9^, ^10^ all patients with FLT3‐ITD mutation without core binding factor (CBF) or adverse risk markers are classified as intermediate risk, regardless of NPM1 co‐mutation or FLT3‐ITD allelic ratio. Second, only in‐frame mutations in the basic leucine zipper (b‐ZIP) domain of CEBPA could be classified as favorable risk, regardless of whether they were monoallelic or biallelic.^11^, ^12^ Finally, *t*(8;16) (p11.2;p13.3)/KAT6A::CREBBP and *t*(3q26.2;v)/MECOM(EVI1) rearrangements, and more myelodysplasia‐related (MR) gene mutations including BCOR, EZH2, SF3B1, SRSF2, STAG2, U2AF1, and ZRSR2 are classified as adverse‐risk markers.

However, the ELN‐2022 risk classification systems remain controversial. Using the Beat AML database cohort, Lachowiez et al^13^ show that the ELN‐2022 guidelines classify patients treated with idarubicin plus cytarabine better, whereas Cancer and Leukemia Group B (CALGB) frontline treatment data show no prognostic advantage for patients with de novo AML compared to ELN‐2017.^14^ Both the German AML Cooperative Group trial data and Spanish real‐world data show that more AML patients are classified as adverse‐risk due to MR‐gene mutation, resulting in inappropriately better survival in the adverse‐risk group of ELN‐2022 compared to ELN‐2017.^15^, ^16^ To date, most of the clinical data to validate the ELN‐2022 risk classification are based on clinical trial results, mostly Caucasian treatment data; some retrospective studies refine the ELN‐2022 risk classification by establishing the “very adverse” group to better clarify the risk groups in the real‐world clinical cohort, showing that more real‐world data are needed to modify the ELN‐2022 risk classification.^17^, ^18^ Herein, we summarized the clinical characteristics and outcomes of 363 patients with primary AML admitted to the First Affiliated Hospital of Anhui Medical University from 2017 to 2021 to evaluate the prognostic value of ELN‐2022 risk classification system compared with ELN‐2017 risk classification system in developing countries.

## 2 PATIENTS AND METHODS

We include newly diagnosed de novo AML patients at our center from January 2017 to December 2021, regardless of the further treatment received. Patients with acute promyelocytic leukemia (APL) and AML with other previous myeloid neoplasms were excluded. All patients were diagnosed according to 2016 World Health Organization (WHO) criteria.^19^ Clinical data including sex, age at diagnosis, hemogram and biochemistry tests at diagnosis, ECOG status at diagnosis, percentage of bone marrow (BM) blasts, myelofibrosis grading, chromosome and next‐generation sequencing (NGS) results, treatment regimen including allogeneic hematopoietic stem‐cell transplantation (allo‐HSCT) results, molecular relapse, and date of survival were included. For patients who received further treatment at other clinical center, we use telephone follow‐up to collect treatment and survival data. The study protocols are in accordance with the Declaration of Helsinki and were approved by the Institutional Review Board of Anhui Medical University.

Cytogenetic analysis was performed on BM cells collected directly or after 24 h of unstimulated culture, and metaphase chromosomes were banded using the trypsin‐Giemsa banding technique. In our center, 20 genes recurrently mutated in myeloid malignancies, including ASXL1, BCOR, CEBPA, DNMT3A, EZH2, FLT3, GATA2, IDH1, IDH2, KIT, KRAS, MLL, NPM1, NRAS, PDGFRA, PHF6, RUNX1, TET2, TP53, and WT1 were analyzed using the Illumina MiSeq instrument (Illumina, San Diego, CA).

We examined associations between ELN genetic risk groups and other patient characteristics by using Fisher's exact test for categorical variables and Wilcoxon rank‐sum test for continuous variables. Survival analysis were performed using the Kaplan–Meier method, and the log‐rank test was used to compare survival between different risk groups. Data were analyzed using IBM SPSS 26.0 and R 4.1.2 (R Development Core Team, Vienna, Austria).

## 3 RESULTS

### 3.1 Baseline characteristics

**FIGURE. 1:**
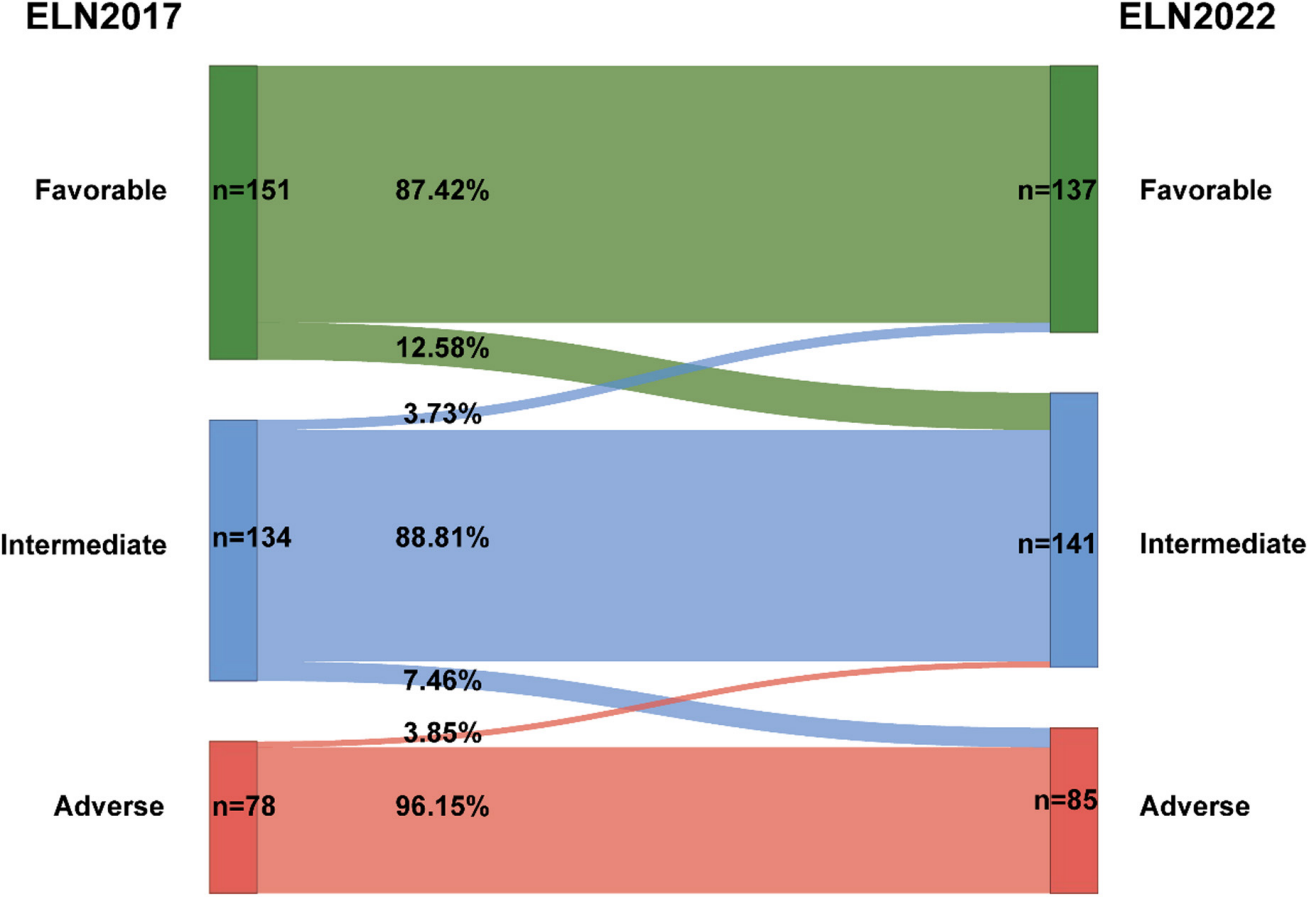
Patient distribution according to European LeukemiaNet (ELN) ‐2017 and ELN‐2022.

From January 2017 to December 2021, 408 patients were diagnosed as de novo AML (excluding APL) in our center, 363 patients have available cytogenetic and NGS data to access ELN‐2022 risk classification. According to ELN‐2022, 137 (37.7%), 141 (38.8%), and 85 (23.5%) patients are classified into favorable, intermediate, and adverse risk groups, respectively, and their basic characteristics are shown in Table 1. The median age of all patients at diagnosis is 52, and becomes older in worse risk groups (favorable vs. intermediate vs. adverse risk groups, 48 vs. 52 vs. 60, *p* < 0.001). Platelet count, white blood cell (WBC) count and BM blasts at diagnosis show differences in ELN‐2022 risk categories (platelet count, *p* = 0.009, WBC count, *p* < 0.001, and BM blasts, *p* = 0.004). Thirty‐seven (10.2%) patients have a change in ELN risk classification compared with ELN‐2017 (Figure 1). In ELN‐2017 favorable risk group, 19 patients whose FLT3‐ITD allelic ratio <50% with NPM1 mutation are reclassified to ELN‐2022 intermediate risk; in ELN‐2017 intermediate risk group, five patients with CEBPA monoallelic bZIP mutation are reclassified to ELN‐2022 favorable risk, 10 patients with MR gene mutations are reclassified to adverse risk; in ELN‐2017 adverse risk group, three patients with FLT3‐ITD allelic ratio >50% are reclassified to ELN‐2022 intermediate risk. In 363 patients available to access ELN‐2022 risk classification, 323 (89.0%) received induction therapy; 271 patients received cytarabine‐based chemotherapy, 52 patients unfit for intensive chemotherapy received hypomethylating agents (HMA)‐based therapy. Thirty‐six (9.9%) patients only received best supportive care, mainly due to early death during hydroxyurea treatment, have serious infection or did not want any therapy due to potential treatment costs, and four (1.1%) patients received treatment in other hospital cannot provide clear induction therapy regimen on telephone.

**TABLE. 1:** Patients characteristics according to ELN‐2022 risk groups.

	Overall *N* = 363	ELN 2022 risk classification			
		Favorable *N* = 137	Intermediate *N* = 141	Adverse *N* = 85	*p*‐value
Age, years					
Median	52	48	52	60	<0.001
Range	14–88	15–86	14–79	22–88	
Age group, *n* (%)					
<60	249	111	97	41	<0.001
≥60	114	26	44	44	
Sex, *n* (%)					
Male	209	73	78	58	0,071
Female	154	64	63	27	
Hemoglobin (g/L)					
Median	73	73	72	72	0.465
Range	21–163	21–138	29–138	26–163	
Platelet count (×10^9^/L)					
Median	34	26	38	45	0.009
Range	2–705	3–225	3–445	2–705	
WBC count (×10^9^/L)					
Median	17.44	19.01	30.39	7.95	<0.001
Range	0.3–370.92	0.3–370.92	0.6–282.07	0.45–305.59	
Bone marrow blasts, %					
Median	54.5	52	61	45	0.004
Range	5–95	5–93	13–95	5.5–91	
Myelofibrosis status^a^					
MF‐0	123	52	43	28	0.161
MF‐1	135	46	56	33	
MF‐2	23	14	4	5	
MF‐3	14	3	7	4	
ECOG status					
0	23	12	9	2	0.146
1	208	85	82	41	
2	84	27	32	25	
3	44	12	16	16	
4	4	1	2	1	
2017 ELN					
Favorable	151	132	19	0	<0.001
Intermediate	134	5	119	10	
Adverse	78	0	3	75	
Induction therapy^a^					
Cytarabine based therapy	271	109	104	57	0.398
HMA based therapy	52	19	19	15	
Best supportive care	36	8	16	12	
Complete remission rate	58.1% (211)	74.4% (102)	53.1% (75)	40.0% (34)	<0.001
Received HSCT	16.3% (59)	18.2% (25)	15.6% (22)	12.9% (11)	0.478
Median overall survival (months)	15.0	27.6	10.6	4.7	<0.001

Abbreviations: HMA, hypomethylating agents; HSCT, hematopoietic stem‐cell transplantation; WBC, white blood cell.

^a^ Myelofibrosis status is unknown in 68 patients; 4 patients cannot provide clear induction therapy regimen.

### 3.2 Outcomes of AML patients according to ELN‐2017 and ELN‐2022 risk classification

**FIGURE. 2:**
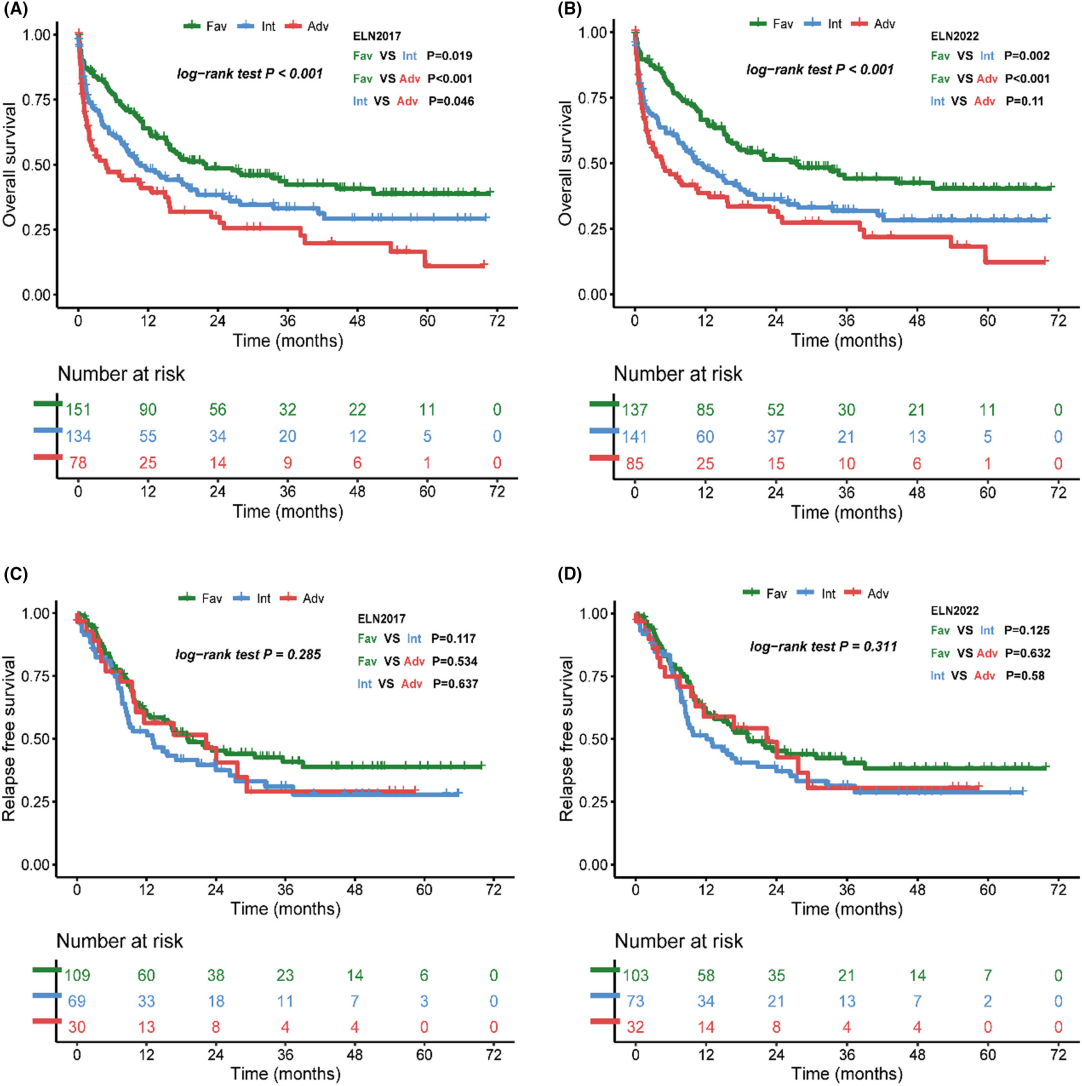
Outcome of patients according to European LeukemiaNet (ELN) ‐2017 and ELN‐2022 risk groups. (A) Overall survival of ELN‐2017 risk classification groups. (B) Overall survival of ELN‐2022 risk classification groups. (C) Relapse‐free survival (RFS) of ELN‐2017 risk classification groups. (D) RFS of ELN‐2022 risk classification groups.

In patients available to access ELN‐2022 risk classification, 211 (58.1%) patients received complete remission (CR), with CR rates in the ELN‐2022 favorable, intermediate and adverse groups of 74.4%, 53.1%, and 40.0%, respectively (*p* < 0.001). For the corresponding ELN‐2017 categories, CR rates were 72.1%, 52.2%, and 41.0%, respectively. After a median follow‐up of 36.9 months, the median overall survival (OS) is 27.6 months, 10.6 months, and 4.7 months in the ELN‐2022 favorable, intermediate and adverse risk groups, compared to 21.9 months, 10.6 months, and 4.8 months in the corresponding ELN‐2017 risk groups. Patients in the ELN‐2022 favorable group have a significantly better OS than those in intermediate and adverse group (*p* = 0.002 and *p* < 0.001, Figure 2B), while there are no statistical differences between the intermediate and adverse groups (*p* = 0.11). However, when patients are classified according to the ELN‐2017 risk classification, we find statistical difference among all risk groups, including intermediate and adverse risk group (*p* = 0.046, Figure 2A). We examined relapse‐free survival (RFS) among patients who achieved CR, but family members of patients who received further therapy and died in other clinical center cannot clearly remember the date of relapse, so there are no statistical differences between ELN‐2017 and ELN‐2022 risk groups (Figure 2C,D). We use ROC curves to evaluate the accuracy of the ELN‐2017 and ELN‐2022 risk classification systems in predicting CR (Figure S1). The area under the curve (AUC) of the ELN‐2017 and ELN‐2022 curves are 0.638 (95%Cl 0.587–0.688) and 0.651(95%Cl 0.600–0.700), respectively. There are no significant statistical differences between the ROC curves of ELN‐2017 and ELN‐2022.

### 3.3 Outcomes classified by induction and post‐remission therapy

**FIGURE. 3:**
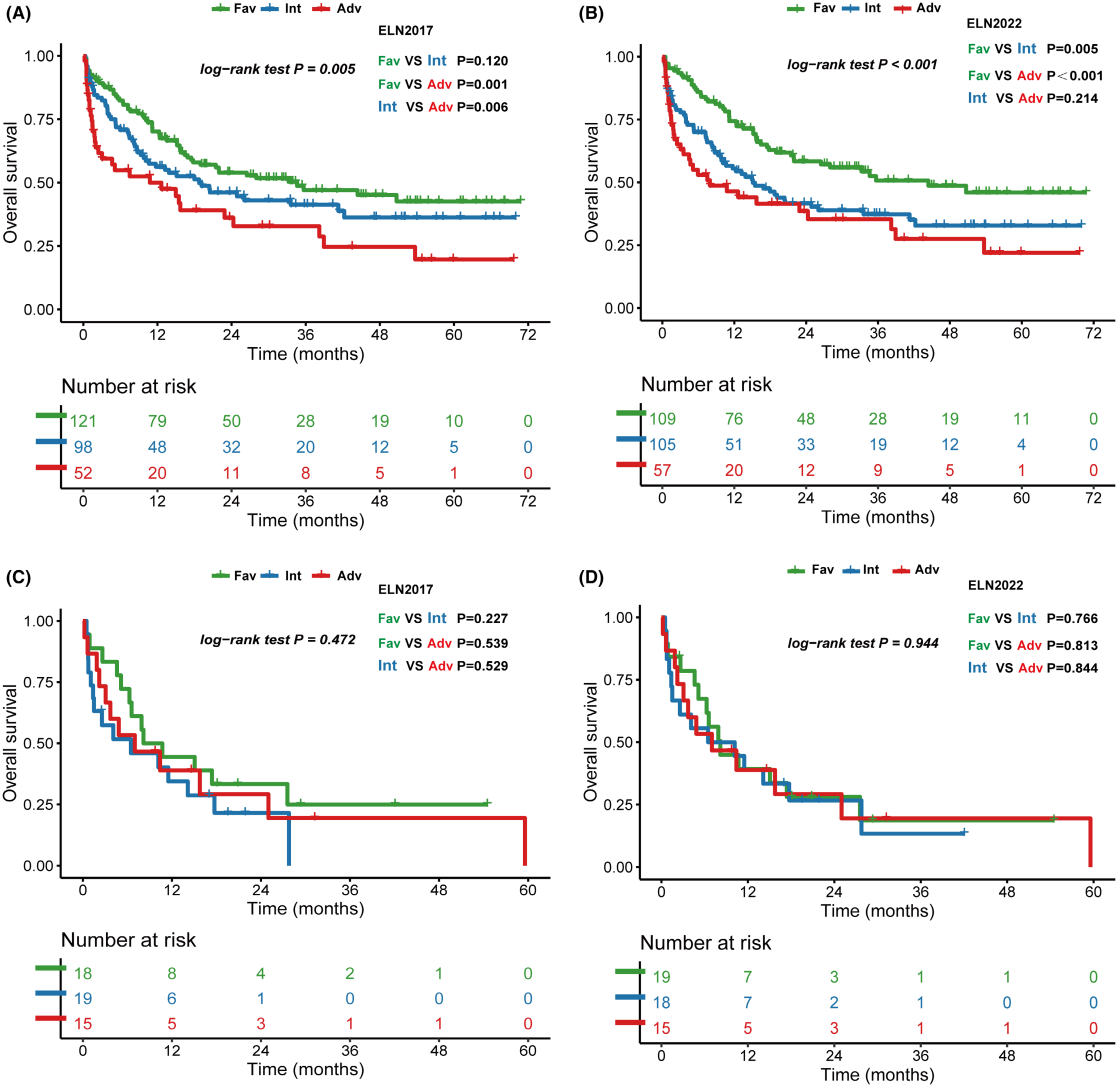
Overall survival of patients by induction therapy received. (A) European LeukemiaNet (ELN) ‐2017 and (B) ELN‐2022 risk groups for patients received cytarabine‐based therapy, (C) ELN‐2017 and (D) ELN‐2022 risk groups for patients received hypomethylating agents (HMA) ‐based therapy.

ELN risk classification is based on clinical trial in which patients received intensive therapy, so we further analyzed OS in patients who received different induction therapies. For 271 patients who received cytarabine‐based induction therapy, ELN‐2017 risk classification cannot clarify the OS of intermediate risk patients with favorable risk group in statistical difference (intermediate vs. favorable, *p* = 0.12, Figure 3A); while in ELN‐2022 risk classification, favorable group have better OS than intermediate and adverse group (*p* = 0.005; *p* < 0.001, Figure 3B), but have no statistical difference between intermediate and adverse risk group (*p* = 0.248, Figure 3B). For 52 patients who received HMA‐based induction therapy, neither ELN‐2017 nor ELN‐2022 risk classification could classify their OS (Figure 3C,D) due to limited sample size.

**FIGURE. 4:**
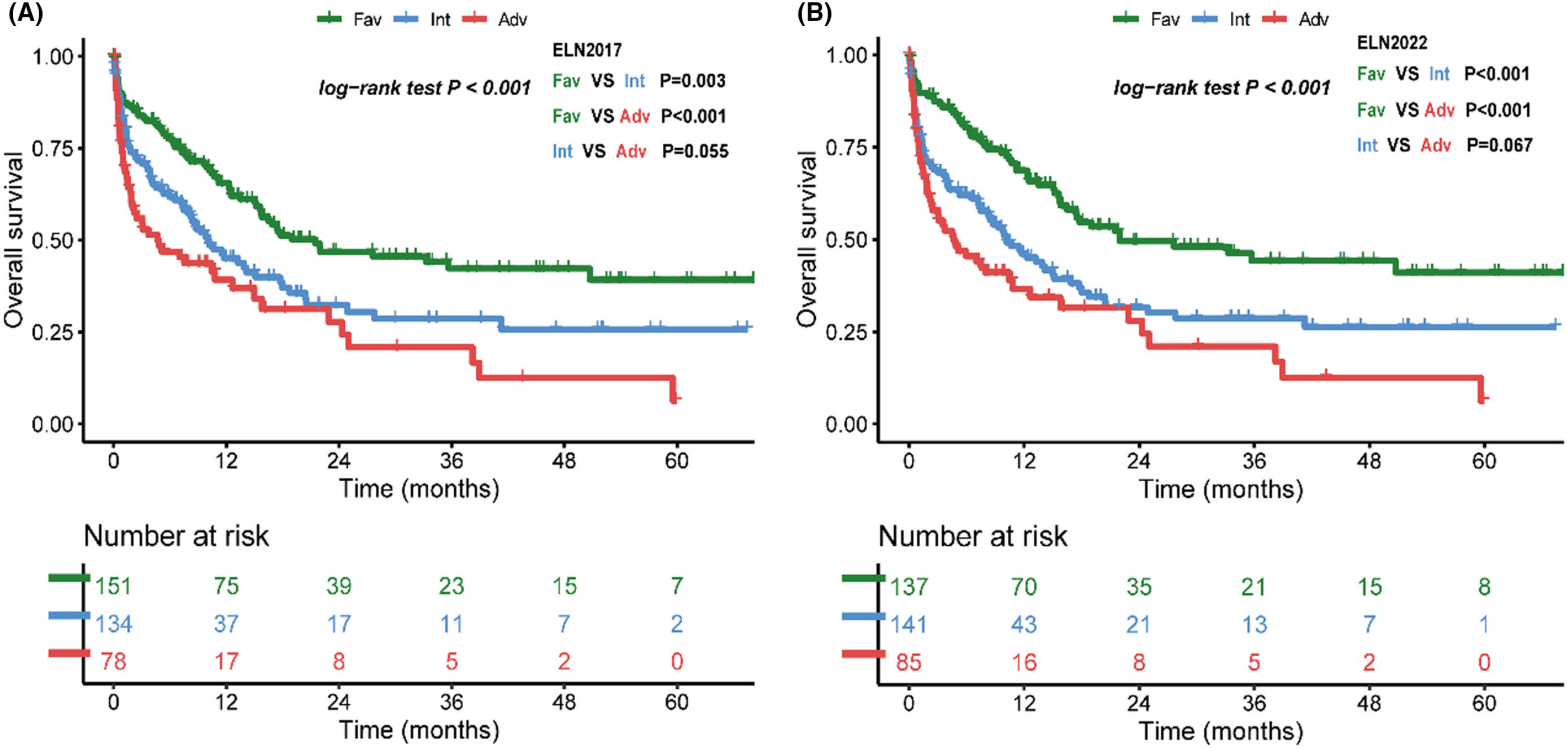
Overall survival of patients when allogeneic hematopoietic stem‐cell transplantation (allo‐HSCT) was censored. (A) European LeukemiaNet (ELN)‐2017 risk groups and (B) ELN‐2022 risk groups.

There are 59 patients (16%) received allo‐HSCT, and their distribution into different ELN‐2022 risk groups is not statistically different (*p* = 0.478, Table.1). As allo‐HSCT would improve the long‐term prognosis,^3^ we additionally evaluated the OS of each risk group when allo‐HSCT was censored. We found that OS of ELN‐2017 and ELN‐2022 favorable groups are both statistically different from intermediate and adverse groups, but neither risk classification system reached statistical difference between intermediate and adverse groups (Figure 4A,B).

### 3.4 Outcome of ELN‐2022 refined subgroups

**FIGURE. 5:**
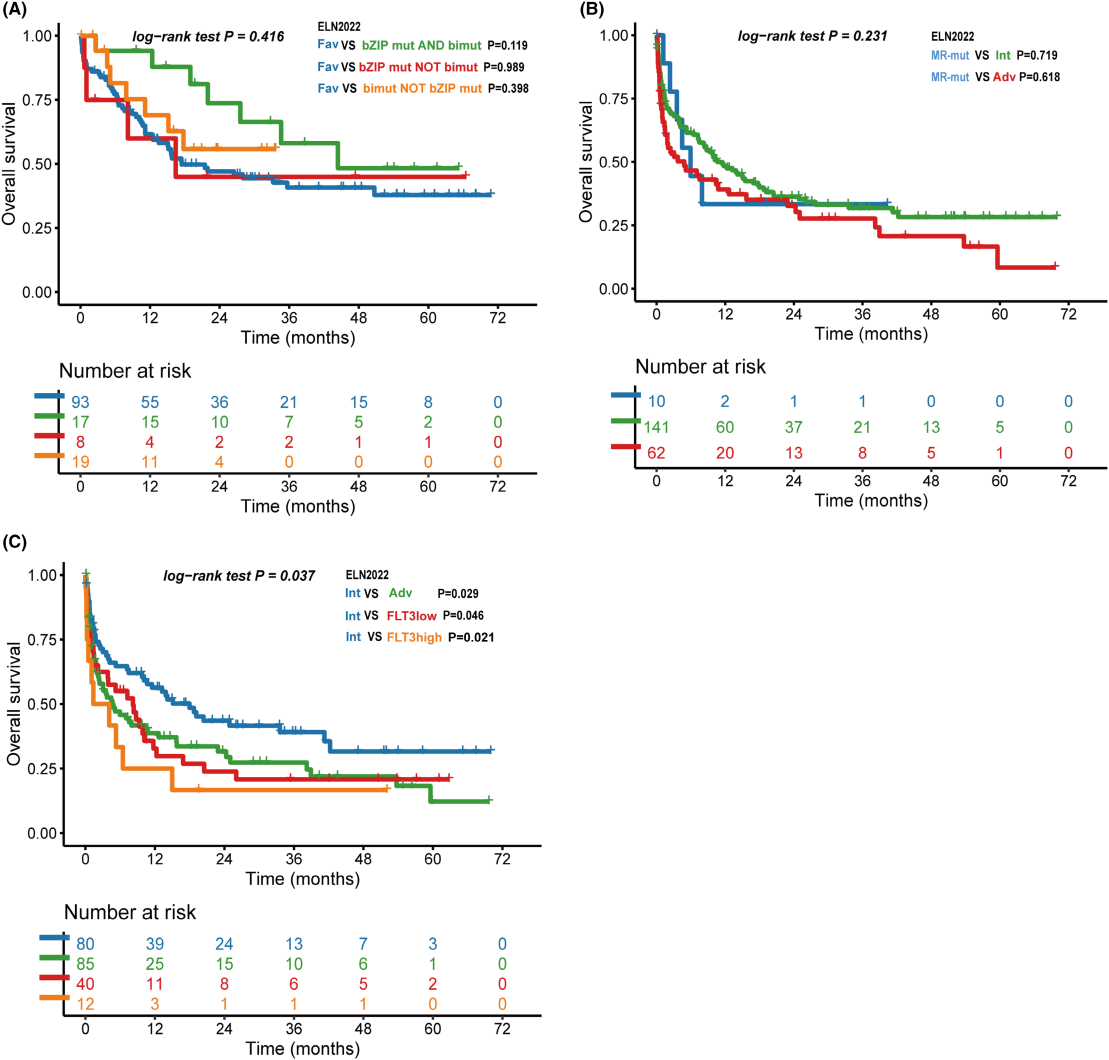
Overall survival of European LeukemiaNet (ELN)‐2022 refined patients. (A) Overall survival of patients with CEBPA mutation, compare with other ELN‐2022 favorable risk patients. (B) Overall survival of patients with myelodysplasia‐related (MR) gene mutation, compare with ELN‐2022 intermediate risk and other ELN‐2022 adverse risk patients. (C) Overall survival of patients with FLT3‐ITD mutation not received FLT3 inhibitor, compare with other ELN‐2022 intermediate risk and adverse risk patients.

We enroll 44 patients with CEBPA mutations without adverse prognostic markers. Among these patients, 17 have a biallelic mutation in the bZIP region, eight have a monoallelic mutation in the bZIP region, and 19 have a biallelic mutation not in the bZIP region. We find no significant statistical differences between these groups compared to ELN‐2022 favorable risk patients without CEBPA mutation, mainly due to the limited number of samples (Figure 5A). Ten patients with MR gene mutation are reclassified from ELN‐2017 intermediate risk groups to ELN‐2022 adverse risk groups. These patients have a similar median OS (5.9 months) with the ELN‐2022 adverse risk group (4.8 months) rather than the ELN‐2022 intermediate risk group (10.6 months), though we find no statistical difference among all groups (Figure 5B).

In our cohort, 61 patients with FLT3‐ITD mutations without adverse prognostic markers are classified as ELN‐2022 intermediated risk. Among these patients, only nine received FLT3 inhibitors (gilteritinib or sorafenib); the remaining 52 patients received chemotherapy alone: 40 have FLT3‐ITD allelic ratio <50%, 12 have allelic ratio ≥50%. These patients unable to receive FLT3 inhibitor did not achieve the same survival outcomes as other ELN‐2022 intermediate risk patients (FLT3‐ITD allelic ratio <50% group vs. intermediate risk group, *p* = 0.046; FLT3‐ITD allelic ratio >50% group vs. intermediate risk group, *p* = 0.021, Figure 5C); instead, both FLT3 mutated groups have no survival difference compare to the ELN‐2022 adverse risk group. Moreover, for the ELN‐2022 intermediate risk group without FLT3‐ITD mutated patients, we find statistical difference to ELN‐2022 adverse risk group on OS (median OS 17.6 months vs. 4.6 months, *p* = 0.029), which is not achieved when FLT3‐ITD mutated patients is included. These results suggest that the ELN‐2022 classification for FLT3‐ITD mutation into intermediate risk may be inappropriate for patients who are unable to acquire FLT3 inhibitors.

### 3.5 Association between ECOG performance status at diagnosis with survival and the ELN‐2022 risk classification

**FIGURE. 6:**
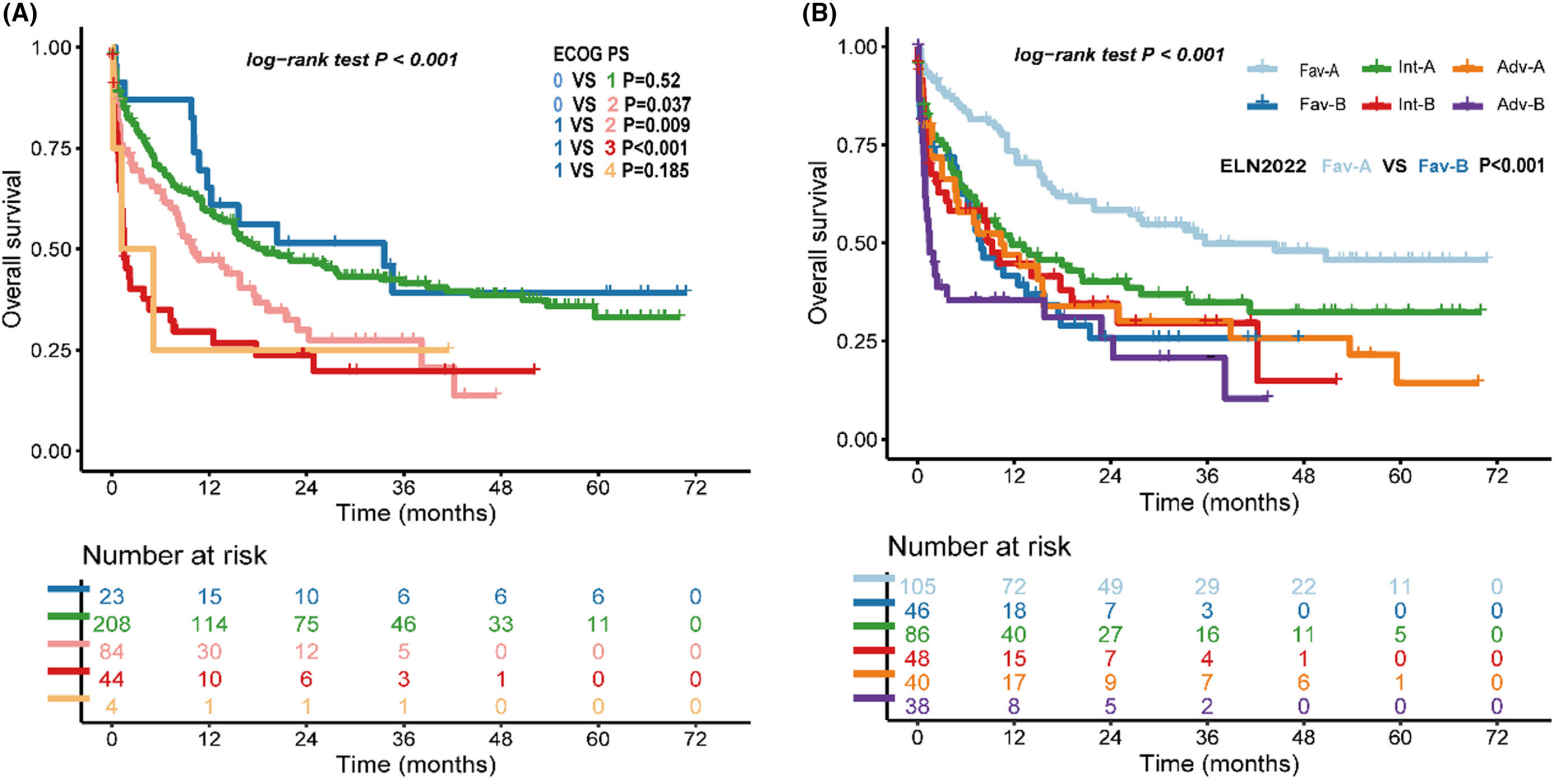
Overall survival according to ECOG status. (A) Overall survival of each ECOG PS. (B) Overall survival of groups combine ECOG PS and European LeukemiaNet (ELN) ‐2022 risk groups, group A = ECOG PS 0–1, group B = ECOG PS 2–4.

We examine the survival data of each ECOG PS group, which show that patients with ECOG PS 0–1 have better survival outcome compared with PS 2–4 (Figure 6A). Combined with the ELN‐2022 risk classification, we divided each risk group into group A (ECOG PS 0–1) and group B (ECOG PS 2–4), and the OS of the ELN‐2022 favorable risk group with PS 0–1 is significantly better than other groups, including the other ELN‐2022 favorable group patients with ECOG PS 2–4 (median survival, 44.4 months vs. 8.2 months, *p* < 0.001, Figure 6B).

## 4 DISCUSSION

The ELN‐2022 risk classification system is an expert panel system based on data from intensively treated patients, and they have indicated that it may need to be modified for patients received inferior intensive therapies.^3^ By using cytogenetic and molecular test results without additional experimental testing, the ELN‐2010 and ELN‐2017 risk classification systems have been widely used worldwide. The ELN‐2022 risk classification system only changes some criteria of risk groups, involving 10% patients in our cohort, so we find no significant differences between the ROC curves of the ELN‐2017 and ELN‐2022 risk classification systems. In our study, we show that the ELN‐2017 risk classification clearly discriminates OS in de novo AML patients and is effective in separating patients in real‐world settings regardless of therapy received, although it does not reach a significant statistical difference as in other cohorts from clinical trials.^5^, ^15^ For the ELN‐2022 risk classification system, favorable risk patients have better survival outcomes compared to ELN‐2017, but there are no significant statistical differences in OS between intermediate and adverse risk patients.

The ELN risk classification system has indicated that it may need to be modified for patients who received inferior intensive therapies. Based on clinical practice, there are still some questions about the ELN risk classification system. Patients age over 60 years old account for 31.4% of our newly diagnosed AML patients, most of whom unable to receive intensive therapy; survival outcomes of clinical trials based on Caucasian data are unclear to fit East Asian people. By classifying patients with different induction therapy received, ELN‐2022 risk classification system is suitable for patients who received cytarabine‐based chemotherapy, but cannot clarify patients who received HMA‐based therapy, which is the same as Sargas^18^ reported. After censoring patients who received allo‐HSCT at the time of transplantation, both ELN‐2017 and ELN‐2022 have no significant statistical differences between intermediate and adverse risk groups. Given that there is no statistical difference among risk groups on induction therapy and HSCT status, these results indicate that treatment regimen may not account for the nonsignificant statistical differences between intermediate and adverse risk groups.

The modification from ELN‐2017 to ELN‐2022 risk classification system in our center patients includes three parts: CEBPA mutated patients; MR gene mutated patients; FLT3‐ITD mutated patients. For CEBPA and MR gene mutated patients, our cohort shows that both modifications are accord with real‐world survival data, which is consistent with other studies.^20^, ^21^, ^22^, ^23^ The outcome of patients with FLT3‐ITD mutation remains controversial.^24^, ^25^, ^26^, ^27^ In ELN‐2022, FLT3‐ITD mutated patients without core binding factor (CBF) or adverse risk markers are all modified as intermediate risk groups, according to the clinical trial results of midostaurin on patients with FLT3‐ITD not NPM1 mutation and MRD in treatment decisions.^3^ However, most patients in China are unable to receive target drugs used in clinical trials prior to the approval by the National Medicine Products Administration (NMPA): in mainland China, midostaurin is not approved yet; the indication for sorafenib does not include AML; and Gilteritinib is not approved for medical use until February 2021. Therefore, most of our FLT3‐ITD mutated patients did not receive FLT3 inhibitor before 2021; although all patients in our center received MRD follow‐up, their OS is not the same as other intermediate risk patients, as a retrospective study from Taiwan, China reported.^17^ Data from our center shows that their survival should be considered as adverse risk groups, as well as their treatment regimen and allo‐HSCT therapy.

ECOG performance status in newly diagnosed patients describes their level of functioning, and affects their treatment and survival,^28^, ^29^ but the relationship between ECOG PS and survival outcome is unclear. The ECOG PS of patients in AML clinical trial is mainly 0–2,^30^ so we wonder whether the ELN risk classification would apply to patients with higher ECOG PS patients. Our data show that patients with ECOG PS 0–1 have better OS than those with ECOG PS 2–4, which is consistent with other real‐world data and prognostic indexes reported.^29^, ^31^ When combining ECOG PS with ELN‐2022 risk classification system, the ELN‐2022 risk classification system does not work well in ECOG PS 2–4 patients; ECOG PS 2–4 patients have worse outcome than others in the ELN‐2022 favorable risk group. As a prognostic index in cytogenetically normal AML including ECOG PS,^31^ ELN‐2022 risk classification system may also need to combine ECOG PS into risk declaration.

The main limitation of our study is the insufficient follow‐up data. Some patients received further treatment in other clinical center due to limited transplantation ward or economic burden of treatment and transportation. In telephone follow‐up of patients died in other center, their family members cannot clearly recall the date of relapse as their death, which makes it difficult for us to obtain high‐quality RFS data. After excluding these patients from RFS, we cannot find significant difference between patients in ELN‐2017 or ELN‐2022 risk groups as OS results, so we do not analysis relapse data in further subgroup analysis. In addition, sequencing of AML recurrent genes was not performed in all newly diagnosed patients before 2017; patients prefer to receive cost‐efficient fusion gene testing at that time, which prevent us from including more patients in our cohort.

In conclusion, our real‐world clinical data show that the ELN‐2022 risk classification system is not superior to the ELN‐2017 version in classifying patients in our center. For patients with FLT3‐ITD mutation but unable to receive FLT3 inhibitor, their risk classification and treatment therapy should be considered as adverse risk group. Based on clinical trial data, ELN‐2022 risk classification system is suitable for ECOG PS 0–1 patients at diagnosis, but not successfully clarify the OS of ECOG PS 2–4 patients. We hope larger cohort based on real‐world data in developing countries could improve the accuracy and applicability of risk classification for newly diagnosed AML patients worldwide.

## AUTHOR CONTRIBUTIONS

**Enbo Chen:** Data curation (equal); formal analysis (equal); writing – original draft (lead). **Changqing Jiao:** Data curation (equal); formal analysis (equal); methodology (equal); project administration (equal); software (equal); writing – review and editing (equal). **Jian Yu:** Data curation (equal). **Yu Gong:** Formal analysis (equal); software (equal). **Duo Jin:** Data curation (equal). **Xiaoyu Ma:** Formal analysis (equal). **Jianling Cui:** Data curation (equal). **Zhonghui Wu:** Data curation (equal). **Junjie Zhou:** Formal analysis (equal). **Haixia Wang:** Data curation (equal). **Bobing Su:** Formal analysis (equal). **Jian Ge:** Conceptualization (lead); funding acquisition (lead); supervision (lead); writing – review and editing (equal).

## CONFLICT OF INTEREST STATEMENT

The authors declare no conflicts of interest.

## ETHICS STATEMENT

The study was approved by the Institutional Review Board of the First Affiliated Hospital of Anhui Medical University, and written informed consent was waived because all research data are based on previous clinical data; our research presents no more than minimal risk of harm to subjects and involves no procedures for which written consent is normally required outside the research context.

## Supporting information


Figure S1:

## Data Availability

The datasets generated during and/or analyzed during the current study are available from the corresponding author on reasonable request.
