# Supplementary material for: Assessment of 2022 European LeukemiaNet risk classification system in real‐world cohort from China
Source: Cancer Med. 2023 Dec 14;12(24):21615–26. doi: 10.1002/cam4.6696 (PMC10757130; doi:10.1002/cam4.6696)
Supplement: Supplementary file 1 — Figure S1: [file CAM4-12-21615-s001.docx]

**Assessment of 2022 European LeukemiaNet risk classification system in real-world cohort from China**

**Enbo Chen^1^, Changqing Jiao^1^, Jian Yu^1^, Yu Gong^1,2^, Duo Jin^1,3^, Xiaoyu Ma^1^, Jianling Cui^1^, Zhonghui Wu^1^, Junjie Zhou^1^, Haixia Wang^1^, Bobing Su^1^ and Jian Ge^1^**^*^

1 Department of Hematology, The First Affiliated Hospital of Anhui Medical University, Hefei, Anhui Province, China

2 Department of Hematology, Chaoyang Hospital, Huainan, Anhui Province, China

3 Department of Hematology, Taihe County People's Hospital, Fuyang, Anhui Province, China

* Correspondence: Jian Ge, gejian52@163.com

Enbo Chen, Changqing Jiao and Jian Yu contributed equally to this work.

**List of supplementary information**

1. Supplementary figure


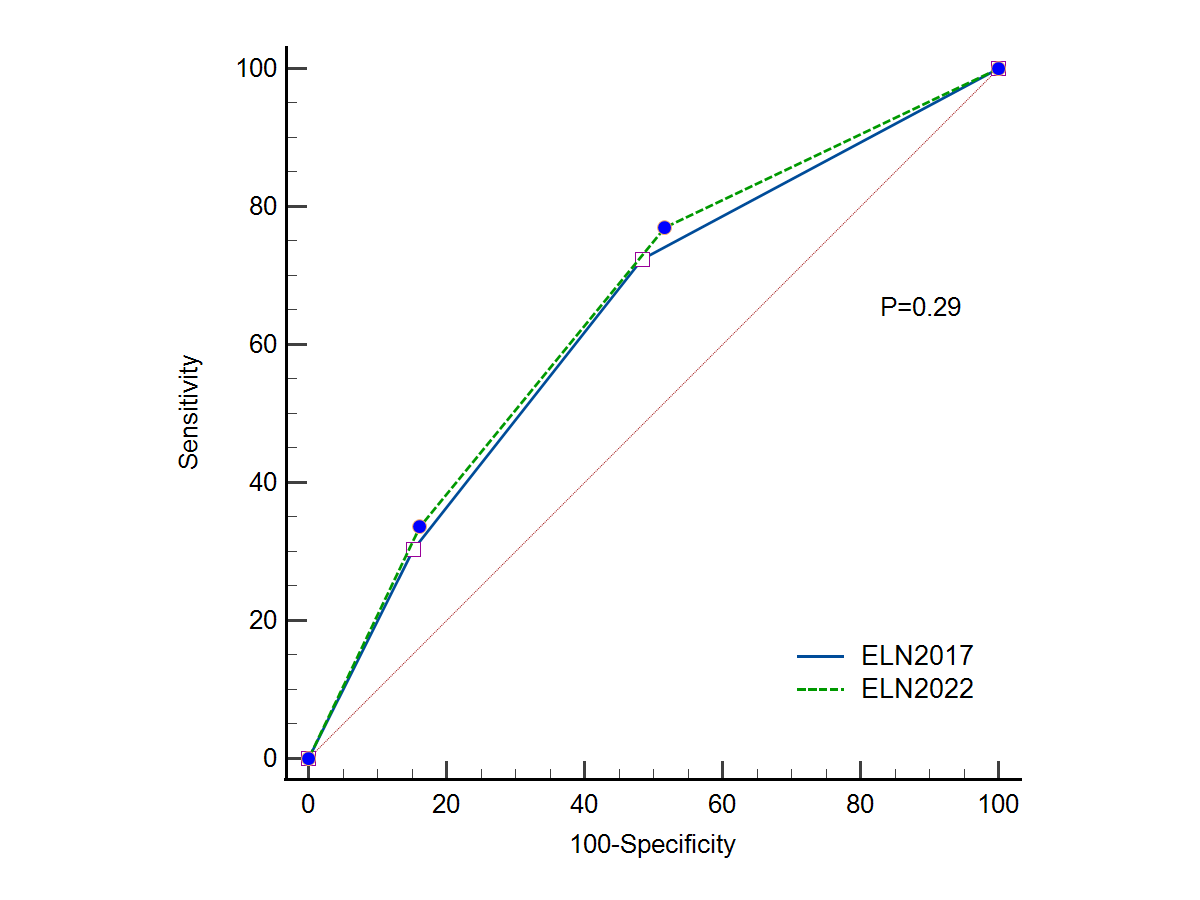


**Supplementary figure.1** ROC curves of ELN-2017 and ELN-2022 risk classification system
